# Neutralization Kinetics and Transport Define the Dermal Decontamination Window for Warfare and Industrial Toxicants

**DOI:** 10.1021/acs.chemrestox.5c00540

**Published:** 2026-04-13

**Authors:** Laurent Simon, Ishita Kulkarni

**Affiliations:** Otto H York Department of Chemical and Materials Engineering, 5965 New Jersey Institute of Technology, Newark New Jersey 07102, United States

## Abstract

Chemical warfare agents that are moderately to highly volatile can readily permeate the skin, posing an acute systemic toxicity risk. Effective decontamination depends on how intervention timing and chemical reactivity jointly shape dermal absorption, evaporation, and neutralization. While earlier finite-dose models characterized dermal uptake, they lacked decontaminant reactivity and failed to quantify how neutralization competes with diffusion and surface clearance. This work presents a unified, dimensionless framework integrating diffusion, evaporation, and reactive neutralization. The system behavior is governed by two dimensionless groups: a surface-loss number (*π_surf_*) that captures combined evaporation and interfacial reaction and a Damköhler number (*Da*) that quantifies bulk neutralization relative to diffusion. Analytical solutions yield characteristic time constants describing the relative rates of absorption, surface clearance, and neutralization. Three distinct kinetic regimes emerge: (i) low *Da* (<3), where slow internal reaction leaves surface loss as the primary clearance mechanism; (ii) intermediate *Da* (3–8), characterized by competitive transport and reaction rates; and (iii) high *Da* (>8), where rapid bulk neutralization renders surface-based interventions progressively less effective. The framework identifies a critical decontamination window during which the applied decontaminant neutralizes the agent faster than it can enter the bloodstream, thereby minimizing systemic exposure. Implemented as open-source Python software, this tool can help emergency responders and regulatory agencies predict decontamination windows for chemical warfare agents and industrial toxicants.

## Graphical abstract

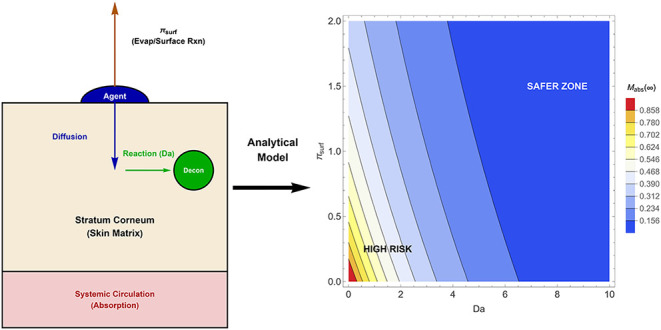

## 1 Introduction

### 1.1 The Challenge of Volatile Agent Dermal Exposure

Under dermal contact, chemical warfare agents (CWAs) such as VX, Sarin, and Novichok can rapidly enter the bloodstream. The window for effective treatment is narrow, typically 15 to 30 min, before extensive systemic absorption occurs. To accurately predict this crucial time frame, sophisticated mathematical models are necessary. These models must account for competing factors such as the rate of agent penetration through the skin, its tendency to evaporate from the surface, and the neutralizing effects of applied countermeasures.

Although current dermal exposure models allow for analysis of these processes, they were not designed to incorporate intervention strategies. Traditionally, dermal exposure assessments have concentrated on evaluating absorption without accounting for potential interventions. Finite-dose models^1,2^ have defined the dynamics of penetration and the evaporation-to-absorption ratios for volatile compounds. Meanwhile, permeability databases^3^ have enabled mechanistic predictions across diverse chemical classes. These contributions, however, do not account for active decontamination measures that alter concentration profiles through *in situ* chemical reactions.

The importance of this gap becomes clear when assessing reactive decontaminants for field use. Reactive Skin Decontamination Lotion (RSDL) has emerged as a frontline countermeasure that effectively neutralizes V- and G-series nerve agents when applied within 15 to 30 min of exposure.^4,5^ Yet systematic reviews^5^ reveal significant variability in effectiveness across agents and application timings. Furthermore, current guidance relies primarily on empirical testing rather than predictive mechanistic models. A quantitative framework linking chemical reactivity, transport properties, and intervention timing to decontamination outcomes remains largely absent.^6^

### 1.2 Quantitative Framework for Decontamination Modeling

To address this gap, this work presents a dimensionless mathematical framework that simultaneously describes the diffusion of volatile organic compounds (VOCs) through the stratum corneum, evaporative surface loss, and reactive neutralization within a unified transport-reaction model. The structure is built on well-established skin barrier models^7^ and finite-dose evaporation frameworks^2,8^ and incorporates first-order neutralization kinetics that represent the reactive mechanism of RSDL.^9^ This enhancement is driven by recent evidence of RSDL efficacy against Novichok agents^9^ and systematic comparisons showing agent-dependent performance variability.^5^

The need for this extension becomes apparent when examining the shortcomings of current techniques. Earlier finite-dose models^1,2,10,11^ successfully predicted absorption and evaporation outcomes but did not incorporate decontaminant reactivity. As a result, they could not describe how neutralization competes with transport or identify the time window during which intervention remains effective.

To enable such predictions, the proposed approach focuses on two dimensionless groups: a Damköhler number (*Da*) representing the ratio of reaction rate to diffusion rate, and a surface loss number (*π_surf_*) that combines evaporation and interfacial reaction. These parameters are computed from experimentally determined properties (diffusion coefficients, reaction rate constants and vapor pressures). Consequently, they can be used to classify decontamination scenarios as diffusion-controlled (low *Da*), reaction-controlled (high *Da*), or a combination of both. While the framework is developed with warfare agents as the primary motivating application, the underlying transport-reaction physics applies broadly to volatile dermal contaminants, including industrial solvents, pesticides, and occupational chemicals. The dimensionless formulation developed in this work enables direct extension to these lower-toxicity scenarios. By adjusting key parameters (diffusion coefficients, reaction rates, and acceptable exposure thresholds), it retains the same analytical structure.

This methodology offers several advantages. It enables the derivation of analytical expressions for absorption and neutralization rates, along with their associated time scales. This structure facilitates the prediction of the decontamination window, a crucial period during which neutralization can outpace systemic uptake. Implemented as open-source Python software, the model allows researchers and emergency planners to evaluate intervention strategies for chemical warfare agents, industrial solvents (e.g., methylene chloride and toluene), and agricultural chemicals (e.g., organophosphate pesticides) without specialized mathematical expertise or custom code development.

## 2 Materials and Methods

This section describes the core components of the unified decontamination model and emphasizes their practical applications. The detailed derivations are provided in the Supporting Information. Here, the focus is on the physical meaning of the dimensionless parameters used to quantify decontamination efficiency, as well as on the software that enables users to apply the model without redeveloping its underlying mathematics.

### 2.1 Problem Statement

Figure 1 illustrates the physical system under consideration. The stratum corneum is depicted as a one-dimensional slab of thickness *h*, with an initial agent deposit in its upper layer (defined as a penetration depth β of 0.1^11^). Three competing mechanisms determine the agent’s fate: (1) inward diffusion toward the systemic circulation, (2) surface loss through evaporation and interfacial reaction and (3) bulk neutralization throughout the skin.

**Figure 1:**
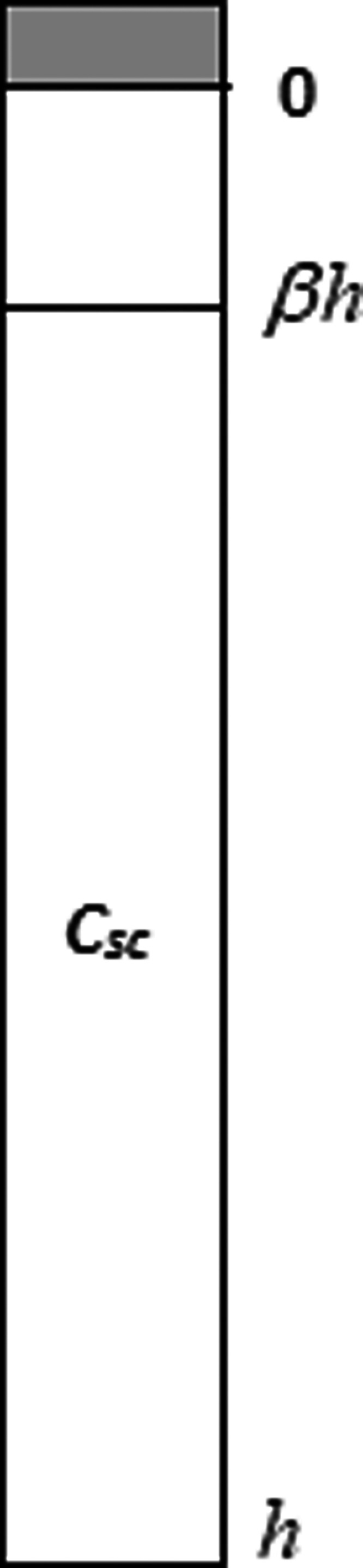
Schematic of the one-dimensional dermal exposure model. The stratum corneum (thickness *h*) initially contains a finite agent deposit (shaded area); β denotes the fraction of the stratum corneum depth over which the agent is initially distributed. The contaminant diffuses toward the systemic circulation (*z* = *h*) while undergoing surface loss (*z* = 0) and bulk neutralization throughout the tissue.

The approach applies to a wide range of agents and exposure scenarios and is based on two dimensionless parameters that can be computed from experimental data. The first is the Damköhler number (*Da* = *k h* ^2^/*D*_SC_), which compares the diffusion to the reaction time scale. *Da* = 1 represents the theoretical threshold where reaction and diffusion time scales are equal. Values below 1 indicate transport-driven behavior, while values above 1 indicate reaction-dominated behavior. More granular depictions can be observed in intermediary zones, as the transition between transport-limited and reaction-limited regimes is a continuous spectrum rather than a sharp cutoff at exactly *Da* = 1. The second parameter is a surface loss coefficient (π_surf_ = κ_evap_ + *Da*_surf_), which represents the combined surface clearance (i.e., evaporation and interfacial reaction) relative to bulk diffusion. For a particular agent-decontaminant combination, *Da* and *π_surf_* are determined from the diffusion coefficient, reaction rate constant, and vapor pressures. These estimates then identify the dominant removal pathway. For more details, please refer to the Supporting Information, Section S2.

### 2.2 Outcome Measures

The model describes three complementary pathways following a dermal dose. The absorbed portion (*M*_abs_) denotes the quantity that enters the bloodstream. The surface loss fraction (*M*_surf_) refers to the portion that is lost due to evaporation or other surface-level reactions. Lastly, the reacted amount (*M*_reac_) represents the amount undergoing chemical reactions within the skin. These three components collectively uphold the principle of mass conservation: *M*_surf_ (∞) + *M*_abs_ (∞) + *M*_reac_ (∞) = 1. Each pathway has an effective time constant *(τ*_*e*ff,abs_, *τ_eff,surf_*, and *τ_eff,reac_*), indicating how long it takes for 98% of that process to complete. The sequence of these time constants provides insight into the decontamination window, which is the period during which neutralization can intercept the diffusing agent before substantial systemic absorption occurs. Analytical expressions for the steady-state mass fractions and the effective time constants, which are functions of *Da*, π_surf_ and β, were derived using Laplace transform methods (Supporting Information, Sections S2–S4).

### 2.3 Computational Implementation

All analytical solutions were implemented as open-source Python software, which makes the tool accessible without requiring users to reproduce the mathematical derivations. The code allows researchers to (1) compute dimensionless parameters (*Da* and π_surf_) using agent characteristics (such as diffusion coefficient and vapor pressure), decontaminant properties (including reaction rate constants), and exposure scenarios (e.g., evaporation rate); (2) calculate the process behavior, including steady-state mass fractions (*M_abs_* (∞), *M_surf_* (∞) and *M_reac_* (∞)), effective time constants (τ_eff,abs_, τ_eff,surf_ and τ_eff,reac_), and time-dependent profiles (*M_abs_* (τ), *M_surf_* (τ) and *M_reac_* (τ)); (3) reproduce all figures in this manuscript and create custom plots for user-specified parameter combinations. Supporting Information (Section S5) and the associated repository provide the full codebase, documentation, and Jupyter notebooks.

## 3 Results

The results shown in Figures 2–7 represent analytical model predictions using parameter ranges derived from chemical warfare agent literature. The Damköhler number range (*Da* = 0–10) spans from diffusion-dominated to reaction-dominated regimes, while the surface loss number range (*π_surf_* = 0.4–2.0) encompasses scenarios where surface-clearance mechanisms contribute significantly to contaminant removal. The experimental validation with VX decontamination represents a diffusion-dominated case (*Da* ≈ 0.88, *π_surf_* ≈ 0.17)^6^ with minimal surface loss (evaporation only, no surface reaction), where surface interventions provide substantial benefit as predicted by the framework. The model yields three characteristic time constants (*τ_eff,surf_*, *τ_eff,abs_* and *τ_eff,reac_*) and three steady-state mass fractions (*M_surf_* *(∞)*, *M_abs_* *(∞)*, and *M*_*reac*_ *(∞)*), each corresponding to a distinct removal pathway. Figures 2–7 systematically examine how these metrics change with *Da* and *π_surf_*, revealing the transition from transport-driven to reaction-dominated behavior.

**Figure 2:**
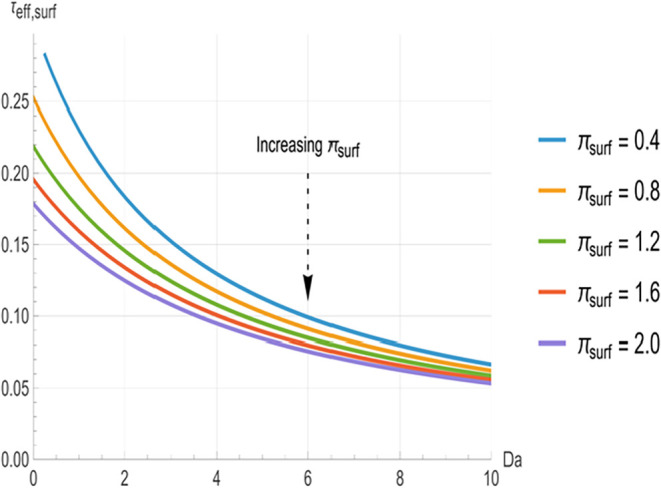
Effective surface clearance time constant (*τ_eff,surf_*) as a function of Damköhler number (*Da*) for various surface loss numbers (*π_surf_* = 0.4, 0.8, 1.2, 1.6, 2.0). Higher *π_surf_* values yield shorter time constants at low *Da*, while the curves converge at *Da* > 8.

This model employs a fully analytical framework designed to provide a deep understanding of underlying mechanisms, rather than statistical fitting to experimental data. Accordingly, it addresses variability through deterministic sensitivity analysis by systematically varying *Da* and *π_surf_* over ranges representative of chemical warfare agents, from persistent agents such as VX to volatile agents such as Sarin. This approach provides a robust map of operational outcomes spanning the full spectrum of experimental variability.

Figures 2, 3, and 4 illustrate the relationship between the effective time constants (*τ_eff,surf_*, *τ_eff,abs_* and τ_eff,reac_) and *Da*. In all cases, the time constants decrease monotonically with increasing *Da*. At low *Da* (<3), the curves are well separated, with higher π_surf_ (indicating greater surface clearance) producing significantly shorter time constants. The greatest separation between the curves occurs in the range of approximately 3 to 8. However, as *Da* increases beyond approximately 8, the curves converge toward common asymptotes, indicating that *π_surf_* variations have a negligible impact at higher reactivity.

**Figure 3:**
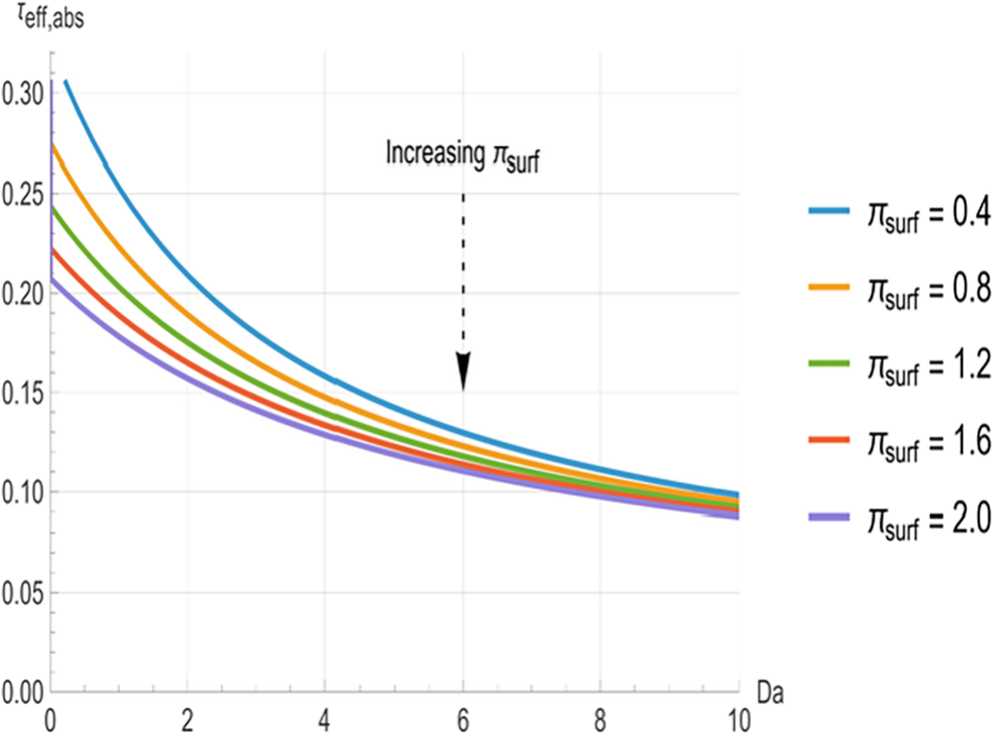
Effective absorption time constant (*τ_eff,abs_*) versus Damköhler number (*Da*) for surface loss numbers ranging from *π_surf_* = 0.4 to 2.0. Absorption times decrease more rapidly with *π_surf_* at low *Da*, while the profiles approach a common plateau at higher *Da*.

**Figure 4:**
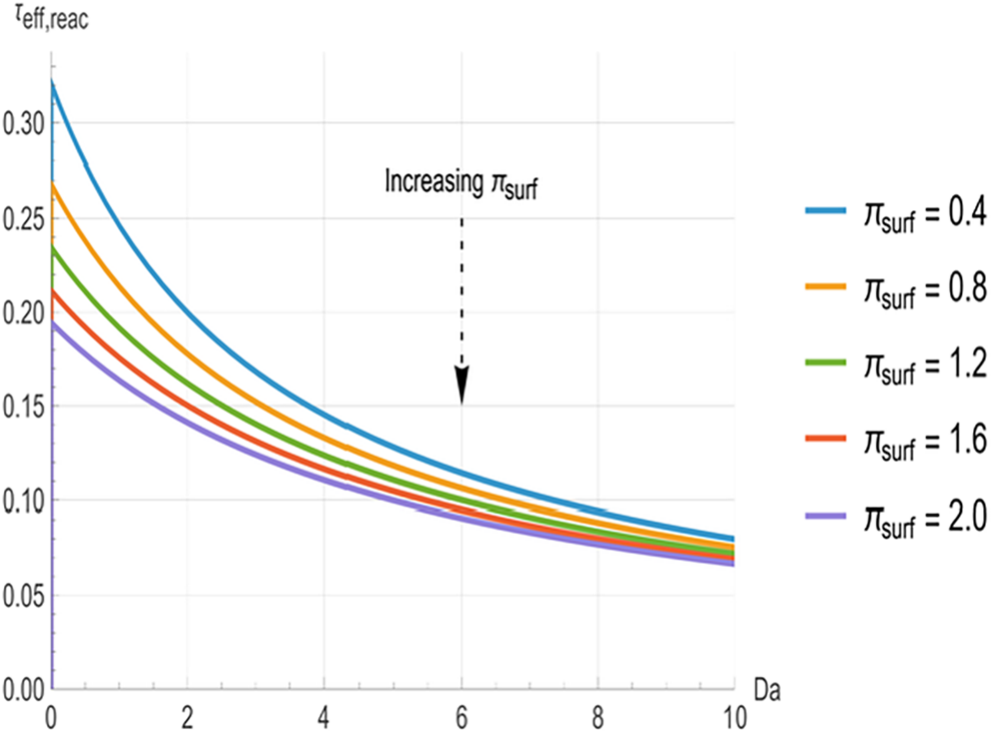
Effective reaction time constant (τ_eff,reac_) as a function of internal Damköhler number (*Da*) for different surface loss numbers (*π_surf_* = 0.4, 0.8, 1.2, 1.6, 2.0). Higher *π_surf_* reduces neutralization times at low *Da*, and the curves converge for *Da* > 8.

Figures 2–4 characterize the kinetics of each removal pathway, while Figures 5–7 show how these mechanisms partition the total deposited mass at equilibrium. The steady-state absorbed mass fraction (*M_abs_* (∞), Figure 5) and surface mass fraction (*M_surf_* (∞), Figure 6) decrease steadily with increasing *Da*, indicating that as bulk reactivity increases, internal neutralization progressively outcompetes surface-loss and absorption pathways. Below *Da* = 6, the curves are well separated, with higher *π_surf_* corresponding to lower *M_abs_* (∞) and higher *M_surf_* (∞). Beyond *Da* ≈ 8, the absorbed fraction approaches a common lower bound where variations in *π_surf_* cause negligible changes. Conversely, the steady-state reacted mass fraction (*M_reac_* (∞), Figure 7) increases monotonically with *Da*, where a smaller *π_surf_* yields a larger *M_reac_* (∞). Unlike the time constants and absorbed fraction, no convergence is observed for *M_reac_* (∞) within this *Da* range. This confirms a sustained dependence on *π_surf_*, as surface processes continue to exert a persistent, though diminishing, influence even in the reaction-control regime.

**Figure 5:**
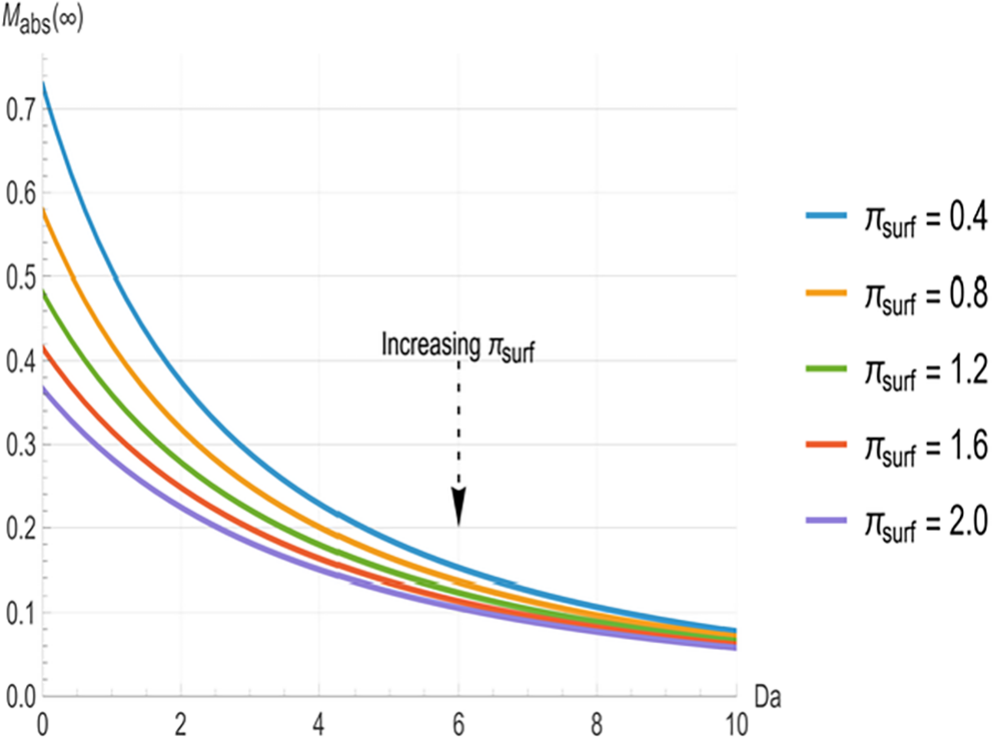
Steady-state absorbed mass fraction (*M_abs_* (∞)) vs internal Damköhler number (*Da*) for various surface loss numbers (*π_surf_*). Higher *π_surf_* values reduce *M_abs_* (∞), particularly at low *Da*, while the curves converge at higher *Da*, indicating minimal dependence on *π_surf_*.

**Figure 6:**
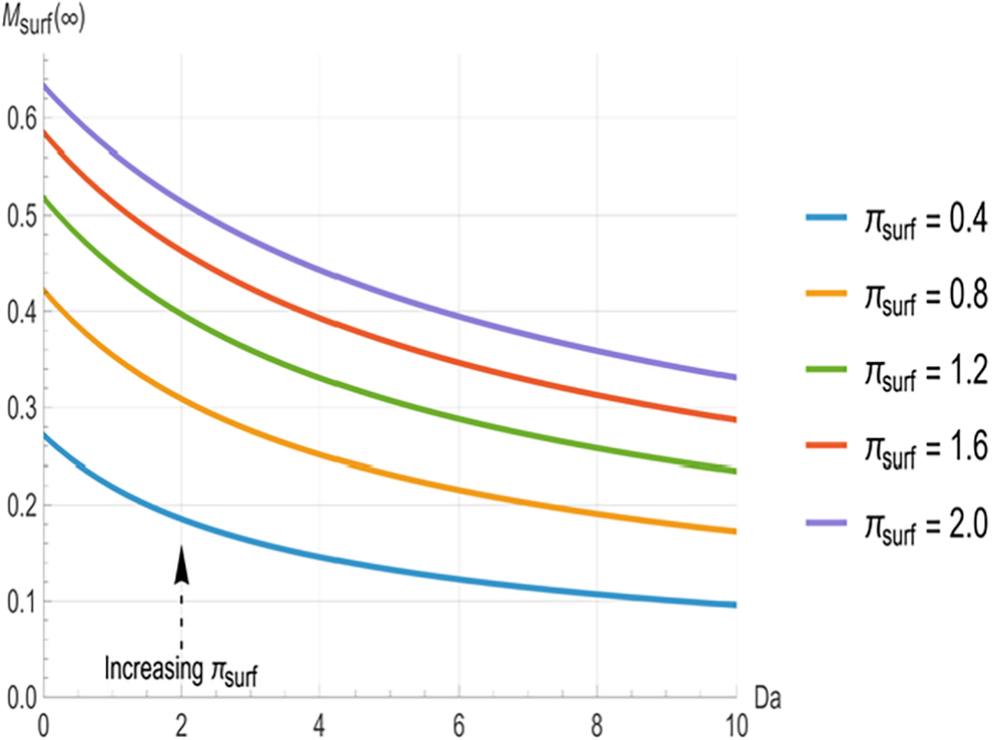
Steady-state surface mass fraction (*M_surf_* (**∞**)) as a function of Damköhler number (*Da*) for surface loss numbers (*π_surf_* = 0.4, 0.8, 1.2, 1.6, 2.0). Higher *π_surf_* yields greater surface removal at all *Da* values, with persistent separation even at high *Da*.

**Figure 7:**
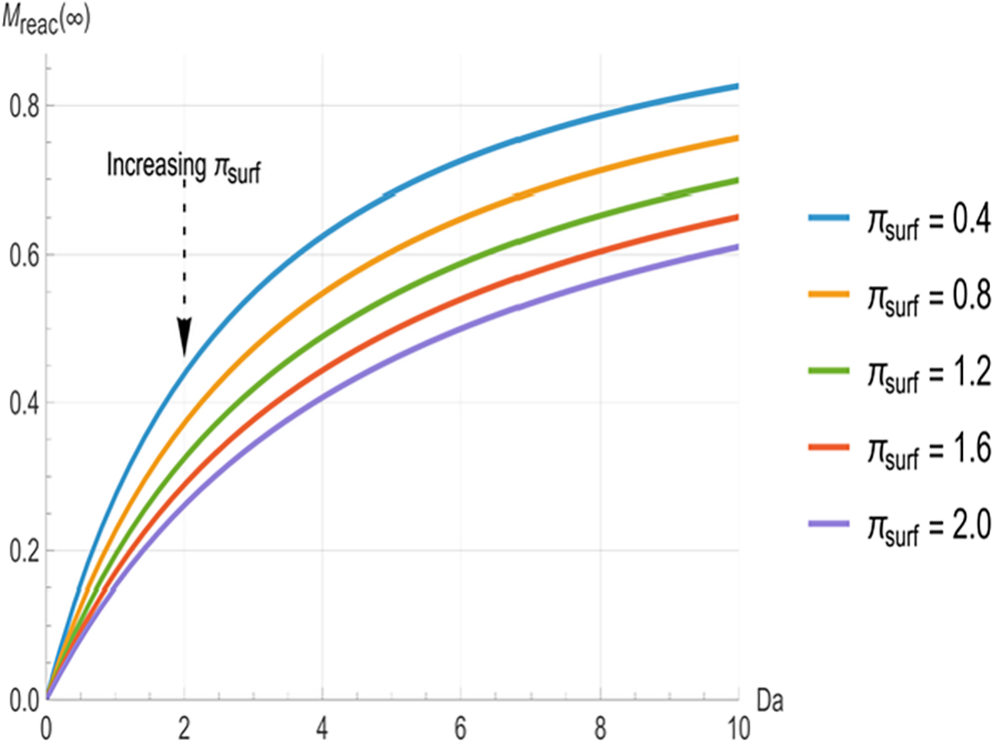
Steady-state reacted mass fraction (*M_reac_* (∞)) as a function of Damköhler number (*Da*) for surface loss numbers (*π_surf_* = 0.4, 0.8, 1.2, 1.6, 2.0). Lower *π_surf_* values yield greater bulk neutralization for all *Da* values.

Figure 8 illustrates the transient behavior of the absorbed, neutralized, and surface mass fractions during toxicant depletion from the skin with and without decontamination. Without decontamination (solid lines), the absorbed fraction rises continuously and dominates the mass balance, while the surface fraction decreases slowly through evaporation alone. When decontamination is applied (dashed lines), the behavior changes markedly. For the simulated conditions (*Da* = 10 and *π_surf_* = 11.2), the surface fraction (*M_surf,d_*) reaches its asymptotic value first, followed by the neutralized fraction (M*_reac,d_*), and finally the absorbed mass fraction (*M_abs,d_*). The surface loss number *π_surf_* = 11.2 is taken from Tabun evaporation data,^6^ representing a highly volatile agent undergoing passive surface clearance. Vertical dashed lines mark four times the effective time constants (4τ_eff_), corresponding to approximately 98% completion of each process. The temporal ordering (4*τ_eff,surf,d_* < 4*τ_eff,reac,d_* < 4*τ_eff,abs,d_*) observed in the plot defines the decontamination window, i.e., the period during which neutralization can intercept the diffusing agent before systemic absorption is complete.

**Figure 8:**
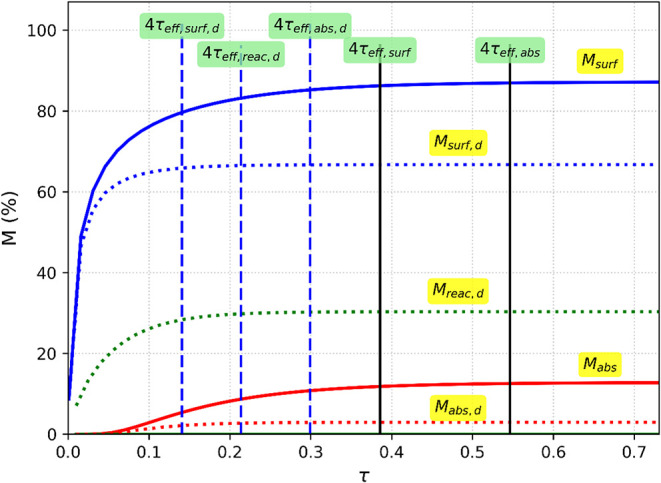
Transient profile of mass fractions for absorption, surface loss, and neutralization in the presence and absence of decontamination. The dashed curves represent the absorbed (*M_abs,d_* (τ)), reacted (*M_reac,d_* (τ)), and surface loss (*M_surf,d_* (τ)) fractions under bulk decontamination, while solid lines denote their values without bulk reaction ((*M_abs_* (τ)) and *M_surf_* (τ)). Vertical dashed lines mark four times the effective time constants **(**4*τ_eff_*) for each process, corresponding to approximately 98% completion. The subscript “*d*” stands for decontamination.

## 4 Discussions

Table 1 outlines the relationship between various parameters and their corresponding outcomes, using the dimensionless framework developed in this study. It illustrates how typical physical quantities, including stratum corneum thickness, agent volatility, and diffusion coefficients, influence the competitive balance between evaporation, surface and bulk neutralization, and systemic uptake. This table shows how changes in physical parameters affect *Da* and *π_surf_*, thereby clarifying the decontamination window and the subsequent kinetic regimes.

**Table 1:** Parameter-to-Outcome Mapping via Dimensionless Framework Analysis

Physical Variable Change	Symbol	Effect on *Da*	Effect on π_surf_	Dominant Mechanism
Thicker Stratum Corneum	↑h	Increases (∝ h^2^)	Increases	Increases the time scale for breakthrough, shifting the balance toward bulk neutralization
More Reactive Lotion (Bulk)	↑k	Increases (∝ k)	No Change	Drives the system toward a bulk reaction-dominated regime
Enhanced Surface Reaction	↑*k*_surf_	No Change	Increases	Surface neutralization dominates over evaporation
Increased Volatility	↑P_vap_	No Change	Increases	Evaporation outpaces penetration, clearing the agent from the surface faster than it can enter the skin
Faster Diffusion	↑D_sc_	Decreases	Decreases	Systemic absorption increases as the decontamination window shrinks.

### 4.1 Kinetic Regimes and Transition Behavior

The results reveal three distinct regimes governed by the balance between transport and reaction. At low *Da* (<3), surface processes (evaporation and interfacial reactions) dominate contaminant removal, and increasing *π_surf_* substantially improves decontamination efficiency. In the intermediate regime (*Da* ≈ 3–8), both mechanisms contribute comparably, making the process highly sensitive to changes in either *π_surf_* or *Da*. At high *Da* (>8), bulk chemical neutralization controls the overall rate, and the system becomes relatively insensitive to surface conditions. This transition from transport-driven to reaction-dominated behavior provides a quantitative framework for optimizing decontamination strategies.

The immediate application of this regime classification is demonstrated using experimentally validated parameters for VX with RSDL.^6^ With a diffusion coefficient *D*_SC_ = 4.3 × 10^–10^ cm^2^/s, a first-order neutralization rate constant *k* = 2.1 × 10^–4^ s^–1^, and a stratum corneum thickness *h* = 13.4 μm, the internal Damköhler number is *Da* = *k h* ^2^/*D*_SC_ ≈ 0.88. This places VX in the low-Da regime (*Da* < 3). Because the internal neutralization rate is too slow to contain the diffusion, the system is diffusion-dominated, making surface processes and early intervention critical. The characteristic diffusion time *h* ^2^/*D*_SC_ ≈ 69.6 min defines the time scale for breakthrough in the absence of intervention. Consistent with this classification, experimental data show that RSDL applied within 5 min reduces cumulative VX absorption by 94.6% (155.7 μg/cm^2^ vs 2,877.0 μg/cm^2^ for untreated controls), while application at 10 min yields 228.4 μg/cm^2^ (92% reduction). Delayed interventions show progressively diminished protection: 30 min (450.1 μg/cm^2^, 84% reduction), 60 min (744.6 μg/cm^2^, 74% reduction), and 120 min (1,340.8 μg/cm^2^, 53% reduction).^6^ The framework thus provides quantitative guidance on intervention timing based on agent-specific transport and reaction properties, with the steep decline in protection factor between 5 and 30 min directly reflecting the diffusion-dominated regime behavior predicted by the model.

Within the intermediate *Da* range, the system is highly responsive to intervention parameters. Minor adjustments in surface conditions, such as increased airflow, solvent volatility, or enhanced surface reaction rates, can yield notable gains in decontamination efficiency. This regime-dependent behavior underscores the importance of optimizing both in-skin chemistry and surface removal mechanisms when designing or evaluating decontamination strategies. Early RSDL application exploits this sensitivity: reactive components neutralize contaminants both at the surface and within the upper stratum corneum while physical removal by evaporation or wiping proceeds simultaneously. Studies show that decontamination delayed to 30–60 min postexposure results in significantly greater skin penetration and lower efficacy.^12^ This experimental observation directly reflects the model’s prediction of a transition to a regime dominated by internal bulk kinetics, where surface interventions provide diminishing returns as the toxicant moves deeper into the tissue, beyond the reach of surface-active decontaminants.

The regime classification has direct implications for decontamination strategies. For agents operating in the high-Da, reaction-dominated regime (*Da* > 8), chemical reactivity is paramount and increasing surface transport offers limited added benefit. In contrast, for VX (calculated *Da* ≈ 0.88 from experimental parameter) and industrial toxicants with similar Da profiles, the system operates in a regime where internal diffusion outpaces bulk neutralization. In this state, the outcome depends largely on surface-clearance mechanisms (i.e., evaporation, wiping, and interfacial neutralization). For such agents, enhancing these surface-level actions can substantially reduce systemic uptake. For both chemical warfare agents and industrial contaminants against which RSDL is only moderately effective, optimal protection is achieved by maximizing surface clearance to ensure the agent is removed or neutralized at the interface before internal transport (diffusion) carries it beyond the reach of the decontaminant.

In practice, optimizing parameters that influence *π_surf_*, such as airflow, solvent volatility and surface flow rate, is most beneficial at low-to-moderate *Da*. This observation is particularly relevant for VX (*Da* ≈ 0.88), which operates squarely in the diffusion-driven regime where surface interventions provide substantial benefit. The same principle applies to volatile industrial solvents and occupational chemicals with similar transport properties. For example, methylene chloride (dichloromethane) and toluene (common degreasing agents with moderate volatility) would benefit from enhanced ventilation and rapid washing in addition to any reactive decontaminant, particularly if their neutralization rates yield *Da* < 3. Similarly, dermal exposure to organophosphate pesticides during agricultural applications could be mitigated through combined physical removal (washing, airflow) and chemical neutralization, with the optimal balance determined by computing *Da* from the pesticide’s diffusion coefficient and the decontaminant’s reaction rate constant. Thors et al. demonstrated that once VX penetrates the deeper skin layers, surface removal becomes ineffective,^13^ consistent with the model prediction that absorbed fraction approaches a limiting value in the reaction-controlled regime. Joosen et al. demonstrated that RSDL application within 30–45 min of exposure markedly reduced systemic uptake in animal models. In contrast, later treatment primarily lowered residual VX in the skin without affecting blood concentrations.^14^ This behavior parallels the model prediction that early decontamination shortens the absorption time constant and reduces *M_abs_* (∞), whereas delayed application occurs after the system approaches its asymptotic state. These findings extend beyond chemical warfare agents to any scenario involving volatile dermal toxicants requiring intervention, including industrial accidents, agricultural exposures, and occupational hygiene applications.

The dependence of *M_surf_* (∞) on *π_surf_* and *Da* (Figure 6) demonstrates the competition between surface depletion and bulk reaction. Under weakly reactive conditions (low *Da*), the process is governed primarily by interfacial depletion, and increasing *π_surf_* shifts the balance toward surface loss. As *Da* grows, however, bulk reaction **i**ncreasingly outcompetes surface loss, reducing *M_surf_* (∞) and its sensitivity to *π_surf_*. Unlike the time constants (Figures 2–4) and absorbed fraction (Figure 5), which converge at high *Da*, *M*_surf_(∞) maintains some dependence on *π_surf_* across the entire *Da* range examined, though the sensitivity diminishes substantially beyond *Da* ≈ 8. In the context of RSDL applications, “surface depletion” corresponds to solvent-assisted lifting/sequestration plus oxime-mediated neutralization at the interface (Dekon-139/DAM in an MPEG–water vehicle).^5^ Thus, higher π_surf_ maps to a stronger interfacial sink from the RSDL formulation, while larger *Da* reflects faster in-skin chemical consumption relative to transport. Empirically, RSDL’s interfacial action rapidly removes/neutralizes VX and other agents,^15^ and increasing reaction dominance (high *Da*) drives the system toward the reaction-controlled limit with diminished sensitivity to interfacial parameters. This behavior is consistent with simulations (Figure 6).

The results in Figure 7 reveal fundamental relationships that have direct implications for the effectiveness of decontamination strategies for both warfare agents and industrial toxicants. The monotonic increase in steady-state reacted mass fraction with *Da* confirms that higher intrinsic reactivity consistently improves decontamination performance, supporting the design rationale of incorporating highly reactive components in systems like RSDL. Systematic reviews demonstrate that RSDL was the most effective decontaminant in nine of 18 experimental models compared with other agents,^5^ validating the importance of chemical reactivity. The persistent inverse relationship between the surface-loss number and the reacted mass fraction indicates that minimizing combined losses from evaporation and surface reaction during contact is critical for optimal performance, a finding that applies equally to volatile industrial solvents and pesticides. Surface loss encompasses both passive evaporation and active neutralization occurring at the skin-decontaminant interface. As illustrated in Figure 7, *M_reac_* remains sensitive to *π_surf_* even under reaction-dominated conditions (*Da* > 8). This continuing dependence arises because surface clearance and bulk reaction are competitive pathways. Molecules intercepted at the surface interface are removed from the system before they can enter the stratum corneum, effectively starving the bulk reaction pathway. Thus, *π_surf_* regulates the total amount of agent accessible to internal neutralization, regardless of the intrinsic reactivity level.

Notably, unlike Figures 5–6, which show convergence or reduced sensitivity at high Da, Figure 7 demonstrates that *M_reac_* (∞) maintains strong dependence on *π_surf_* for the entire *Da* range, with the vertical spacing between curves actually increasing at higher *Da* values, thereby reflecting the direct competition between surface loss and bulk neutralization for the available mass. The model predictions directly translate into field protocols. Field guidance recommends allowing RSDL to remain in contact with contaminated skin for the prescribed 2 min before rinsing,^16^ which aligns with the simulation findings that emphasize the importance of maintaining contact time to maximize the neutralized fraction *M_reac_* over the evaporated/lost fraction *M_surf_*. The concave downward response profiles and lack of curve crossings reflect stable and predictable transport-reaction dynamics. This behavior supports a dual mechanism of physical removal and rapid chemical mixing.^17^ Consequently, this combined approach is essential for mitigating risk from a wide range of threats, including industrial accidents, agricultural exposures and chemical warfare incidents.

### 4.2 Mechanistic Integration

The proposed model offers a unified, first-principles interpretation of dermal exposure and decontamination mechanisms under finite-dose conditions. It simultaneously represents absorption, evaporation, and reactive neutralization within a single quantitative framework. The results demonstrate that mass transport and reactions compete to determine the fate of toxicants, with their relative magnitudes defining the regimes described above. In the low-Damköhler regime (*Da* < 3), internal neutralization is slow relative to transport. Consequently, the independent surface-loss pathway (*π_surf_*) becomes the primary mechanism for clearing the agent from the skin surface. Under these conditions, volatile agents, including many industrial solvents and pesticides, may evaporate or be physically removed faster than they can penetrate or react. As *Da* increases (into the range 3–8), chemical reactions become more important, and both surface and bulk processes contribute comparably. Beyond *Da* ≈ 8, chemical reaction controls the overall process, and the effective time constants converge, marking the transition to a reaction-driven regime. In this regime, systemic absorption risk is governed primarily by reactivity, as decontaminants like RSDL neutralize agents faster than they can diffuse inward. This quantitative characterization of regime transitions provides a rational basis for designing and optimizing highly reactive decontamination systems for chemical warfare agents and toxic industrial compounds. To translate these regime boundaries into actionable guidance, Table 2 provides practical decontamination strategies based on calculated Damköhler number ranges.

**Table 2:** Practical Guidance Based on Damköhler Regime Boundaries

Da Range	Regime	Primary Strategy	Rationale
*Da* < 3	Diffusion-dominated	Maximize surface removal	Bulk reaction in tissue is too slow relative to diffusion; surface removal becomes the primary viable strategy
*Da* = 3–8	Intermediate	Optimize both approaches	Both bulk tissue reaction and surface removal can contribute meaningfully
*Da* > 8	Bulk reaction-dominated	Focus on tissue reaction chemistry	Bulk reaction in tissue is highly effective; surface removal provides limited additional benefit

### 4.3 The Decontamination Window: Definition and Practical Implications

The model quantifies the “decontamination window” as the critical period during which reactive decontaminants can intercept diffusing agents before significant systemic uptake occurs. This window is defined by the temporal ordering of process completion times: 4*τ_eff,surf,d_* < 4*τ_eff,reac,d_* < 4*τ_eff,abs,d_* where the subscript “*d*” denotes decontamination (Figure 8). For low-*Da* systems, e.g., VX (*Da* ≈ 0.88), model calculations confirm this ordering holds for all surface conditions examined.

The observed relationship (4*τ_eff,surf,d_* < 4*τ_eff,reac,d_* < 4*τ_eff,abs,d_*) reflects the spatial hierarchy of the competing pathways. Since surface and bulk reactions begin at the point of application (*z* = 0), they reach their asymptotic values first. In contrast, systemic absorption (*z* = *h*) requires transport through the entire stratum corneum thickness and is the last to finish. This temporal sequence is invariant throughout the explored parameter space because the downstream absorption pathway cannot reach steady state until the upstream surface and bulk pathways have reached completion.

The presence of a reactive decontaminant fundamentally alters removal kinetics, reducing systemic absorption (*M_abs,d_* *<* *M_abs_*) by allowing neutralization to actively compete with inward diffusion. This reveals the basis for the decontamination window: surface and bulk processes are complete while significant diffusion into deeper tissue is still occurring, allowing the intervention to effectively “starve” the absorption process.

Experimental evidence strongly supports the time-dependent effectiveness of this approach. Studies with RSDL against VX confirm this behavior, with Thors et al. showing high efficacy when applied 5–10 min postexposure but significantly reduced effectiveness when delayed beyond 30–60 min, with minimal efficacy at 120 min.^12^ Joosen et al. demonstrated that RSDL decontamination, performed 15 min after exposure, could not prevent progressive blood cholinesterase inhibition. In contrast, decontamination at 90 min still provided some opportunity for treatment, but with substantially reduced effectiveness compared to early intervention.^14^ These observations directly reflect the closure of the decontamination window as the system transitions toward its reaction-controlled steady state.

The same time-dependent urgency applies to industrial toxicants; for example, workers exposed to highly penetrative organophosphate pesticides or corrosive solvents must be decontaminated before the internal diffusion limit is reached. These results provide a quantitative understanding of the period during which reactive decontaminants must be applied to prevent irreversible systemic uptake. For substances with high diffusivity and low volatility, such as VX, Novichok analogs, or nonvolatile industrial toxins, prompt application of a chemical decontaminant is crucial, as they quickly penetrate deeper tissues. Conversely, for volatile compounds such as sulfur mustard or industrial degreasers (e.g., methylene chloride), higher *π_surf_* conditions (e.g., air movement) can accelerate surface clearance even in the absence of high reactivity. From an operational standpoint, the model highlights that effective decontamination for both warfare and industrial threats requires both chemical and physical optimization. Maximizing reactive strength (*Da*) and enhancing surface removal (*π_surf_*) together yield the fastest clearance, underscoring the need for an approach that addresses both kinetics and transport for optimal protection.

To capture the competing effects of internal reaction and surface clearance, Figure 9 presents a two-dimensional phase map of the steady-state absorbed mass fraction for the *Da*-*π_surf_* parameter space. The operational domain is visually divided into distinct risk regions. Scenarios with slow internal neutralization (low *Da*) and poor surface clearance (low π_surf_) create a “High Risk” zone where the majority of the agent is systemically absorbed. Conversely, increasing surface clearance (*π_surf_* >1.5) or internal reaction (*Da* > 6) transitions the system into the “Safer Zone,” where absorption is restricted to less than 20%. This phase map provides practical guidance for decontamination strategies. Chemical exposure scenarios in the high-risk region can be mitigated by increasing *π_surf_* (surface clearance processes) or by increasing *Da* (through more reactive decontaminant formulations). This framework offers a rational basis for tailoring field protocols to specific agent classes, rather than relying on one-size-fits-all contact time and removal procedures for all chemical threats.

**Figure 9:**
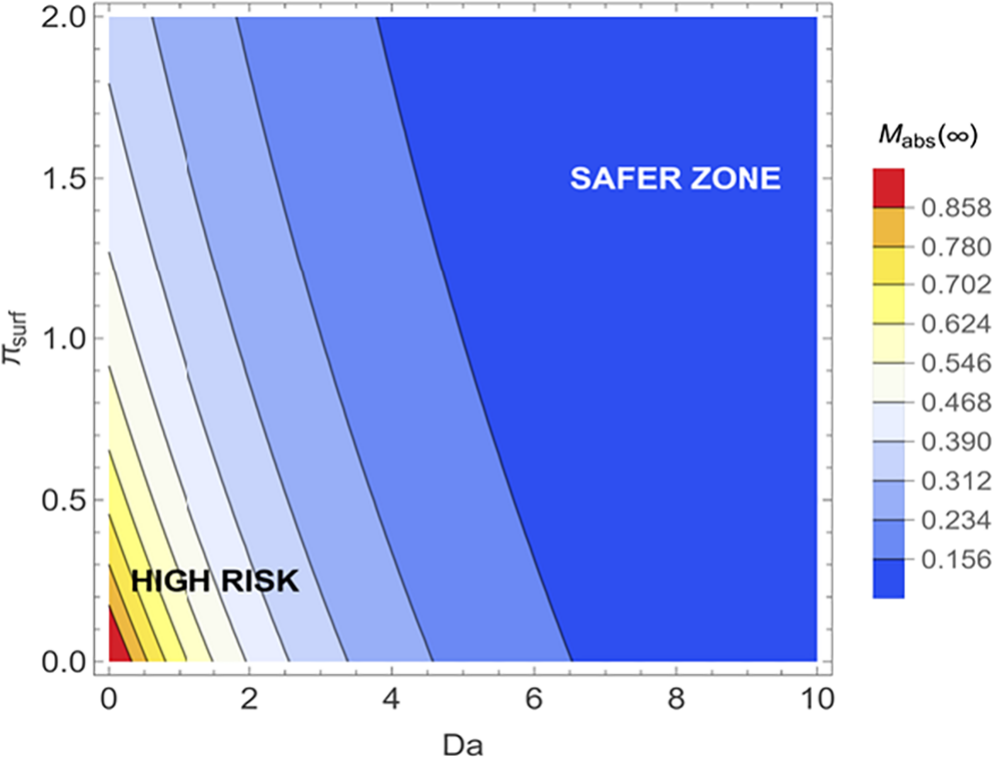
Steady-state absorption risk map across *Da*-*π_surf_* parameter space for chemical decontamination scenarios.

### 4.4 Limitations and Future Directions

The dimensionless approach offers a robust method for evaluating decontamination efficacy. However, its applicability is limited by the underlying assumption of Fickian diffusion and first-order reaction kinetics. This model is especially suitable for nonelectrolytes and lipophilic compounds, such as many organophosphate pesticides and nerve agent analogs, for which transport primarily occurs via diffusion through the lipid-rich stratum corneum. For chemicals that exhibit high ionization, high metabolic activity, or significant tissue binding, the framework remains valuable, using lumped parameters in the core kinetic terms. However, corrosive industrial chemicals fall outside the current scope of this steady-state framework because they damage the skin barrier and alter its thickness or diffusivity in real time. Future iterations of the model could address these factors by introducing time-dependent barrier properties. Nonetheless, the current analysis serves as a foundational tool for addressing the majority of intact-skin exposure scenarios.

The 1D slab model captures the essential mass transport and reaction phenomena and serves as a valid and effective tool for this purpose. However, it simplifies the complex multilayer structure of human skin. It does not explicitly account for the distinct transport properties of the stratum corneum versus the viable epidermis, nor does it include “shunt” pathways, such as penetration through hair follicles and sweat ducts, or compromised skin (e.g., abrasions) that reduce the primary diffusion barrier. Additionally, the model does not consider clothing occlusion, which can suppress evaporation and effectively reduce surface clearance (*π_surf_*). Furthermore, the model assumes constant kinetics. In reality, however, temperature fluctuations are significant in scenarios such as industrial accidents or field exposure, which can affect both the diffusion coefficient and the reaction rate constant. These physiological and environmental factors generally result in faster systemic absorption than predicted, leading to shorter effective decontamination windows.

Moreover uniform RSDL coverage and constant reaction rates are assumed, which may lead to overestimation of neutralization efficiency under nonideal conditions. In industrial settings, where large-scale spills or irregular surfaces (e.g., skin folds or damaged tissue) are common, coverage is often incomplete. To address these limitations, future research could (i) expand the framework to include multilayer geometry to improve the accuracy of depth-dependent concentration profiles, (ii) couple the dimensionless groups (*Da* and *π_surf_*) with experimentally measured temperature dependencies to predict performance in diverse environmental climates and (iii) incorporate stochastic surface coverage to simulate realistic decontaminant deployment. Such refinements would increase the predictive accuracy of the model for a wider range of warfare agents and industrial toxicants in different operational environments.

The VX-RSDL case study serves as an illustrative application demonstrating the model’s mathematical behavior across kinetic regimes governed by *Da* and *π_surf_* using literature-derived parameters, rather than an independent experimental validation. As a result, the current findings should be viewed as a model-based tool for emergency planning and experimental design. Future work must validate the predictive time constants (*τ_eff,surf_*, *τ_eff,reac_* and *τ_eff,abs_*) against controlled *in vitro* (e.g., dynamic Franz cells) and *in vivo* data before the framework can inform definitive clinical guidance.

### 4.5 Software Tool for Practical Application

To make this approach accessible to practitioners without specialized mathematical expertise, all analytical solutions have been implemented as open-source Python software. The complete implementation, including Jupyter notebooks reproducing Figures 2–8, is available at https://github.com/lsimon1/dermal-decontamination-model. Users can modify parameters and regenerate plots for their specific scenarios, systematically varying *Da* and *π_surf_* to visualize regime transitions and identify optimal decontamination strategies without solving the governing differential equations.

This tool directly facilitates the rapid risk assessment and development of safety protocols by emergency responders, occupational hygienists, and regulatory agencies. Users can input the known physical constants for a specific industrial toxicant or chemical warfare agent to determine the “decontamination window” and the relative importance of physical versus chemical interventions. This implementation supports the establishment of evidence-based safety standards by enabling the rapid calculation of decontamination efficacy for warfare agents and industrial toxicants.

## 5 Conclusions

This work presents a unified, dimensionless model that mechanistically describes the dermal decontamination of warfare agents and industrial toxicants under finite-dose conditions by integrating diffusion, evaporation, and chemical reactions into a single predictive framework. The findings delineate three distinct kinetic regimes defined by the Damköhler number (*Da*): at high reactivity (*Da* > 8), bulk chemical kinetics control neutralization and minimize systemic uptake;at intermediate *Da*, both bulk and surface processes compete; whereas at low *Da*, the system is dominated by interfacial processes (i.e., evaporation and surface reaction) governed by *π_surf_*. Experimental validation using VX (*Da* ≈ 0.88) demonstrates quantitative agreement with predictions for the transport-dominated regime; early intervention achieves a 94.6% reduction in absorption, though this efficacy declines to 53% at 120 min. The developed model quantifies the decontamination window, defined by the ordering of characteristic time constants (***τ**_**eff**_*_***, surf,d***_ **<** ***τ**_**eff,reac,d**_* **<** ***τ**_**eff,abs,d**_*), which identifies the critical period during which reactive intervention can intercept agent diffusion before significant systemic absorption occurs. This framework standardizes the analysis of diverse compound classes, including volatile industrial solvents and agricultural pesticides, while the accompanying open-source Python software facilitates rapid, evidence-based risk assessments for emergency responders and occupational hygienists. Ultimately, these findings provide a rigorous quantitative foundation for optimizing the timing and formulation of interventions, translating fundamental transport-reaction principles into objective operational guidance for chemical safety and defense.

## Supplementary Material



## Data Availability

Jupyter notebooks (Figure 2to7git.ipynb and Figure 8git.ipynb) reproducing Figures 2–8, available at https://github.com/lsimon1/dermal-decontamination-model.

## Notes

Nondimensionalization and variable definitions (Section S1), surface-loss parameters (Section S2), Laplace-domain solution and cumulative mass fractions (Section S3) (PDF)
