# Supplementary material for: Neutralization Kinetics and Transport Define the Dermal Decontamination Window for Warfare and Industrial Toxicants
Source: Chem Res Toxicol. 2026 Apr 13;39(5):920–8. doi: 10.1021/acs.chemrestox.5c00540 (PMC13188160; doi:10.1021/acs.chemrestox.5c00540)
Supplement: Supplementary file 1 [file tx5c00540_si_001.pdf]

Supporting Information

Neutralization Kinetics and Transport Define the Dermal Decontamination Window for Warfare and Industrial Toxicants

Laurent Simon* **and Ishita Kulkarni**

Otto H York Department of Chemical and Materials Engineering

New Jersey Institute of Technology, Newark NJ 07102, USA

*Corresponding author. Tel.: +1 973 596 3572; fax: +1 973 596 8436.

E-mail address: laurent.simon@njit.edu (L. Simon)

Table of Contents

Section	Title	Page
S1	Nondimensionalization and variable definitions	1
S1.1	Dimensional governing equation and boundary conditions	1
S1.2	Dimensionless variables	3
S1.3	Dimensionless governing equation and Damköhler number	4
S1.4	Conversion back to dimensional quantities	5
S2	Surface-loss parameters	6
S2.1	Evaporative contribution	7
S2.2	Surface Damköhler number	8
S3	Laplace-domain solution and cumulative mass fractions	8
S3.1	Laplace domain formulation	8
S3.2	Fluxes and cumulative amount of contaminant absorbed and removed	10
S3.3	Effective time constants	11
S3.4	Non-first-order kinetics	12
References		13

S1. Nondimensionalization and variable definitions

S1.1 Dimensional governing equation and boundary conditions

The outer skin layer (stratum corneum) is represented as a planar slab of thickness h (cm), with spatial coordinate z measured from the exposed surface ($z=0$) to the base of the stratum corneum ($z=h$). The dimensional concentration of the contaminant in the stratum corneum is denoted $C_{\mathrm{SC}}(z,t)$ (mg/mL), and time is denoted t (h).

Transport and in-skin neutralization are described by a diffusion–reaction equation of the form

$$\frac{\partial C_{\mathrm{SC}}(z,t)}{\partial t} = D_{\mathrm{SC}} \frac{\partial^2 C_{\mathrm{SC}}(z,t)}{\partial z^2} - k\ C_{\mathrm{SC}}(z,t), \quad 0 < z < h,\ t > 0, \tag{S1}$$

where $D_{\mathrm{SC}}\ (cm^2/h)$ is the effective diffusion coefficient in the stratum corneum and $k\ (h^{-1})$ is the first-order (or pseudo–first-order) rate constant for neutralization in the presence of RSDL. The initial condition corresponds to a finite deposit confined to the superficial portion of the stratum corneum:

$$C_{\mathrm{SC}}(z,0) = \begin{cases} C_0 & 0 \le z < \beta\ h \\ 0 & \beta\ h \le z \le h \end{cases}, \quad 0 < \beta \le 1 \tag{S2}$$

where C_0 (mg/mL) is the initial concentration in the contaminated region and β is the fractional penetration depth of the initial deposit. The total initial mass per unit area present in the stratum corneum is

$$m_0 = \int_0^h C_{\mathrm{SC}}(z,0)dz = \int_0^{\beta h} C_0\ dz = \beta\ C_0\ h. \tag{S3}$$

The quantity m_0 (mg/cm^2) is used as the reference mass scale in the nondimensionalization. At the exposed skin surface ($z = 0$), the contaminant is removed by parallel processes of evaporation and surface reaction with RSDL. The dimensional flux leaving the skin is denoted $J_{\text{surf}}(t)$ (mg/cm^2/h), and the boundary condition is written

$$-D_{\text{SC}} \frac{\partial C_{\text{SC}}}{\partial z}\bigg|_{z=0} = k_{surf} C_{\text{SC}}\left(0,t\right), \quad t > 0. \tag{S4}$$

for the surface flux (evaporation + surface reaction). At the base of the stratum corneum ($z = h$), a perfect sink boundary condition is imposed,

$$C_{\text{SC}}(h,t) = 0, \quad t > 0, \tag{S5}$$

representing rapid clearance into the viable epidermis and dermis. Having established the dimensional problem, we now introduce a dimensionless formulation to identify the key controlling parameters.

S1.2 Dimensionless variables

Dimensionless variables are introduced to reduce the number of independent parameters and to identify the dominant dimensionless groups that control system behavior. The following scalings are used:

$$\xi = \frac{z}{h}, \quad \tau = \frac{D_{\text{SC}}\ t}{h^2}, \quad C(\xi,\tau) = \frac{C_{\text{SC}}(z,t)\ h}{m_0}. \tag{S6}$$

Here, ξ is the dimensionless depth, τ is the dimensionless time, and $C(\xi,\tau)$ is the dimensionless concentration in the stratum corneum. The choice of scaling for C normalizes the total initial mass:

$$\int_0^1 C(\xi,0)\, d\xi = \frac{h}{m_0}\int_0^h C_{SC}\left(z,0\right) dz = \frac{m_0}{m_0} = 1 \tag{S7}$$

so that the total dimensionless mass in the slab equals unity at $\tau = 0$. Using the definition of ξ and $C(\xi,\tau)$ in eq. (S6), the initial condition in Eq. (S2) becomes

$$C\left(\xi,0\right) = \begin{cases} 1 & 0 \le \xi < \beta \\ 0 & \beta \le \xi \le 1 \end{cases},\ 0 < \beta \le 1 \tag{S8}$$

With the dimensionless variables and initial condition established, we now derive the dimensionless governing equation.

S1.3 Dimensionless governing equation and Damköhler number

Substitution of eq. (S6) into eq. (S1) yields, after simplification,

$$\frac{\partial C(\xi,\tau)}{\partial \tau} = \frac{\partial^2 C(\xi,\tau)}{\partial \xi^2} - \mathrm{Da}\, C(\xi,\tau), \quad 0 < \xi < 1,\ \tau > 0, \tag{S9}$$

where $\mathrm{Da} = k\, h^2 / D_{\mathrm{SC}}$ is the internal Damköhler number. This dimensionless group compares the characteristic diffusion time h^2 / D_{SC} to the characteristic reaction time $1/k$ in the stratum corneum. When Da >> 1, neutralization is fast relative to diffusion (reaction-limited transport), whereas Da << 1 indicates diffusion-limited behavior with slow neutralization.

The boundary conditions in eqs. (S4)–(S5) transform to

$$-\left.\frac{\partial C}{\partial \xi}\right|_{\xi=0} = \pi_{\mathrm{surf}} C(0,\tau), \quad \tau > 0, \tag{S10}$$

and

$$C(1,\tau) = 0, \quad \tau > 0, \tag{S11}$$

Here π_{surf} is the dimensionless surface-loss number,

$$\pi_{surf} = \kappa_{evap} + Da_{surf}, \tag{S12}$$

which combines the contributions from evaporation and surface reaction at the skin surface (Section S2). Equations (S9)–(S11), together with the initial condition in eq (S8), constitute the complete dimensionless formulation stated in the main text. This formulation depends on three dimensionless parameters: the internal Damköhler number (Da), the surface-loss number (π_{surf}), and the initial penetration depth (β).

S1.4 Conversion back to dimensional quantities

For comparison with experimental measurements and practical application, the dimensionless results must be converted back to dimensional quantities. The dimensionless solution $C(x,\tau)$ can be mapped back to dimensional concentrations and fluxes using the definitions in Eq. (S6). The dimensional concentration profile in the stratum corneum is obtained as

$$C_{\mathrm{SC}}(z,t) = \frac{m_0}{h}\, C\left(\frac{z}{h}, \frac{D_{\mathrm{SC}} t}{h^2}\right). \tag{S13}$$

The diffusive flux at the base of the stratum corneum ($z = h$) is

$$J_{\text{abs}}(t) = -D_{\text{SC}} \frac{\partial C_{\text{SC}}}{\partial z}\bigg|_{z=h} = -\frac{m_0 D_{\text{SC}}}{h^2} \frac{\partial C}{\partial \xi}\bigg|_{\xi=1,\ \tau(t')}, \tag{S14}$$

which represents the absorbed flux into the viable tissue. The cumulative absorbed mass fraction up to time τ can be written in terms of the dimensionless flux as

$$M_{\text{abs}}(\tau) = \frac{1}{m_0} \int_0^t J_{\text{abs}}(t'), dt' = -\frac{D_{\text{SC}}}{h^2} \int_0^\tau \frac{\partial C}{\partial \xi}\bigg|_{\xi=1,\ \tau'} dt' = -\frac{D_{\text{SC}}}{h^2} \frac{h^2}{D_{\text{SC}}} \int_0^\tau \frac{\partial C}{\partial \xi}\bigg|_{\xi=1,\ \tau'} d\tau' = -\int_0^\tau \frac{\partial C}{\partial \xi}\bigg|_{\xi=1,\ \tau'}. \tag{S15}$$

Analogous expressions can be written for the cumulative neutralized fraction $M_{\text{reac}}(\tau)$ (from volume integration of the Da·C reaction term) and the cumulative surface-loss mass fraction $M_{\text{surf}}(\tau)$ (from time integration of the surface flux at $\xi = 0$). These fractions satisfy the mass balance $M_{surf}(\infty) + M_{abs}(\infty) + M_{reac}(\infty) = 1$, ensuring complete mass accountability. The dimensionless formulation therefore allows concentration profiles, fluxes, and mass fractions to be computed compactly and then converted back to dimensional units using the scaling relations in Eq. (S6).

S2. Surface-loss parameters

The dimensionless surface-loss number π_{surf} introduced in Eq. (S11) plays a critical role in determining decontamination effectiveness. We now detail how π_{surf} is calculated from physicochemical properties and RSDL reaction kinetics.

At the skin surface, contaminant removal occurs through two parallel pathways: evaporation into air and surface reaction with the decontaminant. In the dimensionless boundary condition

$$-\left.\frac{\partial C}{\partial \xi}\right|_{\xi=0} = \pi_{\text{surf}} C(0,\tau), \quad \tau > 0, \tag{S16}$$

the dimensionless surface-loss number,

$$\pi_{surf} = \kappa_{evap} + Da_{surf}, \tag{S17}$$

where κ_{evap} represents the evaporative loss and Da_{surf} denotes the surface neutralization. Each contribution is calculated independently from physicochemical properties as detailed below.

S2.1. Evaporative contribution

The evaporative contribution κ_{evap} can be estimated from physicochemical properties [1]

$$\kappa_{evap} = \frac{h k_{evap} \rho}{D_{SC} C_{sat}} \tag{S18}$$

Using the relationship between gas-phase and skin permeability [1], this expression can be rewritten as

$$\kappa_{evap} = \frac{k_g P_{vap} MW}{RT} \frac{1}{k_p S_w}. \tag{S19}$$

Here, k_{evap} is the evaporation mass transfer coefficient (cm/h); ρ is the toxicant density (mg/mL); C_{sat} is the solubility in the skin (mg/mL); k_g is the gas-phase mass transfer coefficient (cm/h); and k_p is the permeability coefficient (cm/h), which represents the rate at which a chemical penetrates the skin [2].

The thermodynamic parameters are: R, the gas constant (62.37 mL·Torr/K·mmol); T, the absolute temperature (≈ 298 K); P_{vap}, the compound's vapor pressure (Torr); S_w, the solubility in water (mg/mL); and MW, the molecular weight (g/mol). Similarly, Da_{surf} is the surface Damköhler number given by

$$Da_{surf} = \frac{k_{surf} h}{D_{SC}}, \tag{S20}$$

where k_{surf} (cm/h) is the first-order reaction rate at the surface.

S2.2 Surface Damköhler number

Combining the evaporative contribution (Eq. S18) and surface reaction contribution (Eq. S19) yields the total surface-loss number. Substitution of eqs. (S18)–(S19) into eq. (S17) gives the combined dimensionless surface-loss number,

$$\pi_{surf} = \frac{k_{surf} h}{D_{SC}} + \frac{k_g P_{vap} MW}{k_p S_w RT} \tag{S21}$$

which summarizes the competing contributions of evaporation and surface reaction to contaminant removal at the exposed skin surface. Large values of π_{surf} (>> 1) indicate rapid surface removal relative to diffusion, leading to a depletion boundary layer near the skin surface. Conversely, small values (π_{surf} << 1) result in near-uniform concentration profiles with minimal surface effects.

S3. Laplace-domain solution and cumulative mass fractions

S3.1 Laplace domain formulation

To obtain analytical expressions for the cumulative mass fractions and characteristic timescales, we solve Eqs. (S8)–(S11) using Laplace transform methods. This approach converts the time-dependent PDE into an algebraic problem that yields closed-form expressions for absorption, surface loss, and neutralization rates. While the mathematical details are provided for completeness, practitioners may proceed directly to the results in Section S3.2. This framework also facilitates the calculation of dynamic metrics, such as the time required to reach steady state. Taking the Laplace transform with respect to dimensionless time τ (with transform variable σ), Eq. (S9) becomes an ordinary differential equation:

$$(\sigma + Da)\bar{C}(\xi,\sigma) = \frac{\partial^2 \bar{C}}{\partial \xi^2} + C(\xi,0) \tag{S22}$$

where $\bar{C}(\xi,\sigma) = \int_0^\infty C(\xi,\tau)\, e^{-s\tau} d\tau$ is the Laplace transform of the concentration. The transform variable σ is related to the dimensional Laplace variable s by $\sigma = sh^2/D_{sc}$. Equation (S22) is a second-order ODE whose general solution involves exponential functions. Defining $\lambda = \sqrt{\sigma + Da}$ for compactness, the solutions in the two domains are:

$$\bar{C}_1(\xi,\sigma) = A_1 e^{\lambda\xi} + B_1 e^{-\lambda\xi} + \frac{1}{\sigma + Da}, \quad 0 \le \xi \le \beta \tag{S23}$$

and

$$\bar{C}_2(\xi,\sigma) = A_2 e^{\lambda\xi} + B_2 e^{-\lambda\xi}, \quad \beta < \xi \le 1 \tag{S24}$$

The four constants (A_1, A_2, B_1, B_2) are determined by applying: (1) the boundary conditions at $\xi = 0$ and $\xi = 1$ from Eqs. (S10)–(S11), and (2) continuity of concentration and flux at the interface $\xi = \beta$. The algebraic details are omitted for brevity. The Laplace-domain solution enables efficient calculation of both the cumulative mass fractions (Section S3.2) and the characteristic decontamination timescales (Section S3.3).

S3.2 Fluxes and cumulative amount of contaminant absorbed and removed

The model quantifies three removal pathways: (1) surface loss through evaporation and surface reaction, (2) absorption into viable tissue, and (3) neutralization within the stratum corneum. In the Laplace domain (denoted by overbars), the cumulative mass fractions for each pathway are calculated from the transformed concentration gradients:

$$\bar{M}_{surf}(\sigma) = \frac{1}{\sigma}\bar{J}_{surf}(\sigma) = \frac{1}{\sigma}\left(\frac{\partial \bar{C}_1}{\partial \xi}\right)\Bigg|_{\xi=0}, \tag{S25}$$

$$\bar{M}_{abs}(\sigma) = \frac{1}{\sigma}\bar{J}_{abs}(\sigma) = -\frac{1}{\sigma}\left(\frac{\partial \bar{C}_2}{\partial \xi}\right)\Bigg|_{\xi=1} \tag{S26}$$

and

$$\bar{R}_{bulk}(\sigma) = Da\left[\int_0^{f_{dep}} \bar{C}_1(\xi,\sigma)\,d\xi + \int_{f_{dep}}^{1} \bar{C}_2(\xi,\sigma)\,d\xi\right], \tag{S27}$$

respectively. Applying the final value theorem to the Laplace-domain expressions yields the steady-state mass fractions ($\tau \rightarrow \infty$):

$$M_{surf}(\infty)=\frac{e^{-\sqrt{Da}(1+\beta)}\left(e^{2\sqrt{Da}}-e^{\sqrt{Da}\beta}\right)\left(-1+e^{\sqrt{Da}\beta}\right)\pi_{surf}}{2\sqrt{Da}\beta\left(\sqrt{Da}\cosh\left[\sqrt{Da}\right]+\pi_{surf}\sinh\left[\sqrt{Da}\right]\right)}, \quad \text{(S28)}$$

$$M_{abs}(\infty)=\frac{\pi_{surf}(-1+\cosh[\sqrt{Da}\beta])+\sqrt{Da}\sinh[\sqrt{Da}\beta]}{Da\beta\cosh[\sqrt{Da}]+\sqrt{Da}\,\pi_{surf}\beta\sinh[\sqrt{Da}]}, \quad \text{(S29)}$$

$$M_{reac}(\infty)=\frac{e^{-2\sqrt{Da}\beta}\begin{pmatrix}-e^{\sqrt{Da}\beta}\left(-1+e^{\sqrt{Da}}\right)\left(-1+e^{\sqrt{Da}\beta}\right)\left(e^{\sqrt{Da}}+e^{\sqrt{Da}\beta}\right)\pi_{surf}+\\ Da\,e^{2\sqrt{Da}\beta}\left(1+e^{2\sqrt{Da}}\right)\beta+\\ \sqrt{Da}\left(e^{\sqrt{Da}(1+\beta)}-e^{\sqrt{Da}(1+3\beta)}-e^{2\sqrt{Da}\beta}\,\pi_{surf}\beta+e^{2\sqrt{Da}(1+\beta)}\,\pi_{surf}\beta\right)\end{pmatrix}}{\sqrt{Da}\left(\sqrt{Da}-\pi_{surf}+e^{2\sqrt{Da}}\left(\sqrt{Da}+\pi_{surf}\right)\right)\beta} \quad \text{(S30)}$$

These expressions, while algebraically complex, depend only on the three dimensionless parameters (Da, π_{surf}, β) and can be rapidly evaluated for any chemical-decontaminant combination. Figures 5-7 in the main text presents $M_{abs}(\infty)$, $M_{surf}(\infty)$ and $M_{reac}(\infty)$ as functions of Da and π_{surf} for representative decontamination scenarios.

S3.3 Effective time constants

To quantify decontamination speed, we compute characteristic time constants for each removal pathway. The effective time constant τ_{eff} represents the average residence time before a molecule exits via that pathway [6]. This is analogous to a reaction half-life, but accounts for the full temporal distribution of the process. Based on standard methods for characterizing first-order processes [6], the effective time constant for each pathway is calculated as the first moment of its flux distribution. In the Laplace domain, this reduces to:

$$\tau_{eff} = \lim_{\sigma\to 0}\left(\frac{m(\infty)}{\sigma^2} + \frac{d\bar{M}(\sigma)}{d\sigma}\right)\left[\lim_{\sigma\to 0}\left(\frac{m(\infty)}{\sigma} - \bar{M}(\sigma)\right)\right]^{-1} \tag{S31}$$

This expression is evaluated separately for absorption $\left(M_{abs}(\infty)\right)$, surface loss $\left(M_{surf}(\infty)\right)$, and neutralization $\left(M_{reac}(\infty)\right)$ to yield $\tau_{eff,abs}$, $\tau_{eff,surf}$ and $\tau_{eff,reac}$, respectively. Each time constant quantifies a distinct removal mechanism: $\tau_{eff,abs}$ for absorption into viable tissue, $\tau_{eff,surf}$ for surface clearance (evaporation + surface reaction), and $\tau_{eff,reac}$ for in-skin neutralization by RSDL. The critical decontamination metric is the ratio $\tau_{eff,reac}/\tau_{eff,abs}$: when less than 1, RSDL neutralizes the contaminant faster than it can penetrate to systemic circulation. Following standard practice for first-order kinetics [7, 8], we define process completion as $4\tau_{eff}$ (≈98% conversion). Figures 2-4 in the main text show how $\tau_{eff,surf}$, $\tau_{eff,reac}$ and $\tau_{eff,abs}$ vary with RSDL concentration.

S3.4 Non-first-order kinetics

The analytical framework assumes pseudo-first-order kinetics, valid when the decontaminant is in large excess. To demonstrate how bimolecular kinetics would modify the system, consider the governing equation:

$$\frac{\partial C_{\mathrm{SC}}(z,t)}{\partial t} = D_{\mathrm{SC}}\frac{\partial^2 C_{\mathrm{SC}}(z,t)}{\partial z^2} - k\ C_{\mathrm{SC}}(z,t)C_{\mathrm{SC,d}}(z,t), \quad 0 < z < h,\ t > 0, \tag{S32}$$

In this case, the Damköhler number becomes

$$Da = \frac{k\ h^2 C_{SC,d,0}}{D_{SC}} \quad \text{(S33)}$$

where $C_{SC,d,0}$ is the initial decontaminant concentration applied to the skin surface. Partial coverage would be more accurately addressed using a 2-D representation. Other representations of the nonlinearity would change Eq. (S33). Due to the nonlinear nature of the system, we cannot obtain closed-form expressions for the effective time constants using the Laplace transform technique described above.

References

1. Kasting, G.B. and M.A. Miller, *Kinetics of finite dose absorption through skin 2: volatile compounds.* J Pharm Sci, 2006. **95**(2): p. 268-80.
2. Frasch, H.F. and A.L. Bunge, *The transient dermal exposure II: post-exposure absorption and evaporation of volatile compounds.* Journal of pharmaceutical sciences, 2015. **104**(4): p. 1499-1507.
3. Abate, J. and P.P. Valkó, *Multi-precision Laplace transform inversion.* International Journal for Numerical Methods in Engineering, 2004. **60**(5): p. 979-993.
4. Simon, L. and J. Ospina, *Closed-form solutions for drug transport through controlled-release devices in two and three dimensions*. 2015, Hoboken, New Jersey: John Wiley & Sons Inc. p.
5. Ferreira, J.A., et al., *Flux tracking in drug delivery.* Applied Mathematical Modelling, 2011. **35**(10): p. 4684-4696.
6. Collins, R., *The choice of an effective time constant for diffusive processes in finite systems (Thermal conduction and sputtering examples).* Journal of Physics D: Applied Physics, 1980. **13**(11): p. 1935.
7. Simon, L., *Control of biological and drug-delivery systems for chemical, biomedical, and pharmaceutical engineering*. 2013, Hoboken, N.J.: Wiley. xv, 366 p.
8. Smith, C.A. and A.B. Corripio, *Principles and practice of automatic process control*. 3rd ed. 2006, Hoboken, NJ: Wiley. xvi, 563 p.
